# Treatment of Fevers by Salines

**Published:** 1844-04

**Authors:** 


*Treatment of Fevers by Salines.*—In the "Lancet" for December 14, 1839, some very interesting results on the use of salines are given by Dr. Jordan Lynch. He practised in the worst districts of London, and states that his successes, after employing the following treatment, exceeded his most sanguine expectations. After premising an emetic, and a brisk purge of calomel and rhubarb, or jalap, he gave a solution of three drachms of common salt to the pint of water in the twenty-four hours, the patient drinking largely of cold spring water, adding to the mixture a drachm of muriatic acid as the symptoms improved, with effervescing soda powders till convalescence was complete, supporting the strength with beef tea and porter. The acid effectually checked the diarrhœa. Out of 97 cases not one died, and recovery, he says, took place in as many days as it required weeks on the ordinary plan.

Dr. Copland, in his elaborate article "Fever," par. 596, says, "The chloride of soda is a valuable medicine in all the typhoid forms of fever when judiciously prescribed;" and Chomel, who gave it an extensive trial, states that it has proved more successful in low fevers than any other means, when perseveringly employed. Drs. Graves and Stokes also think highly of it in petechial fever.

Dr. Wilson, of the Middlesex Hospital, adopted Dr. Stevens' saline treatment, with great advantage, during the prevalence of petechial fever in 1837. The patients were all put into a warm bath and washed with soap, the head shaved, and cold applied if necessary. The following powder was given in water every four hours: R. Carbonate of soda, half a drachm; chloride of sodium, 1 scruple; chlorate of potass, 6 grains. Mix.

If this were refused, a drachm of the chlorate of potash in a quart of water was given for drink in the 24 hours. In some severe typhoid cases, where active treatment was inadmissible, in addition to wine and beef tea, Dr. Graves gave carbonate of soda, one scruple; nitrate of potash, ten grains, every three hours, with great success.

Dr. Furnival, in his work on consumption and scrofula, says, "In the middle or even later periods of typhus, I must bear testimony to the great efficacy of large doses of the sesquicarbonate of soda alone, every four hours, either in water or some other tonic effusion. It is surprising how soon the tongue will clean, and the collapse give way."

Dr. Bright speaks favorably of a similar plan, and the common effervescing draughts, prescribed as simple refrigerants, may be more actively useful than the prescriber suspects.

Among the German writers there is extensive evidence in favor of the hydro-chlorate of ammonia in putrid adynamic fevers, and a very general preference has been attached by writers of all classes to combinations in which chlorine plays a part. Indeed, the compound recommended by Dr. Stevens is probably resolved in the stomach into the muriates of soda and potash. The nitrate and chlorate of potass are also particularly deserving of trial.

The above practical testimonies in favor of the saline treatment of fever are more than sufficient to entitle the subject to the student's serious consideration; though the value of such treatment is practically demonstrated, the theory is not free from much painful obscurity, but some additional insight into the possible modus operandi of such remedies may be gleaned from Professor Liebig's researches on the influence of soda on the decomposition of our tissues and in the formation of bile."—*Braithwaite's Med. Retrospect, No.* 8, *p.* 20.

